# The complete mitochondrial genome of *Rhynocoris altaicus* Kiritshenko, 1926 (Hemiptera: Heteroptera: Reduviidae)

**DOI:** 10.1080/23802359.2026.2739028

**Published:** 2026-09-29

**Authors:** Miao-Miao Chen, Yi-Neng Zhang, Tong Li, Zhi-Ming Zhang, Jian-Feng Qiu, Hui-Lin Huang, Ren Chen

**Affiliations:** ^a^ School of Habitat Environment, Quanzhou Vocational and Technical University, Quanzhou, China; ^b^ College of Forestry, Fujian Agriculture and Forestry University, Fuzhou, China; ^c^ Forest Pest Control and Quarantine Station, Geology and Mineral Resources Management and Disaster Prevention and Mitigation Section, Minhou County Bureau of Natural Resources and Planning, Fuzhou, China; ^d^ Putian Customs Comprehensive Technical Service Center, Fuzhou Customs District P.R. China, Putian, China

**Keywords:** *Rhynocoris altaicus*, assassin bug, complete mitochondrial genome, phylogenetic tree

## Abstract

In this study, we assembled and characterized the complete mitochondrial genome of *Rhynocoris altaicus* Kiritshenko, 1926, a predatory assassin bug distributed in the eastern Palearctic that feeds mainly on lepidopteran pests. The circular mitogenome is 16,177 bp with 70.20% A+T, comprising the typical set of 37 mitochondrial genes (13 protein-coding genes (PCGs), 22 transfer RNAs, and two ribosomal RNAs), and a 1326 bp D-loop. Phylogenetic analysis based on 13 PCGs recovered *Rhynocoris* as sister to *Sphedanolestes* within Harpactorinae. The mitogenome has been deposited in GenBank as PQ613804. It provides molecular data for taxonomy, phylogenetics, and conservation of Reduviidae.

## Introduction

As the most species-rich subfamily of assassin bugs, Harpactorinae (Hemiptera: Reduviidae) comprises over 2000 species distributed across 300 genera worldwide (Wang et al. 2023). Recent phylogenetic studies have led to a revised tribal classification, with the subfamily now formally recognized as comprising five monophyletic lineages: Apiomerini, Bactrodini, Dicrotelini, Ectinoderini, and Harpactorini (Masonick et al. 2025). Members of Harpactorinae exhibit remarkable ecological niche partitioning, as exemplified by *Rhynocoris altaicus* Kiritshenko, 1926, a common species in northern China and Mongolia, characterized by a single red annular marking on each femur basally and with significant potential for biological control as a predator of lepidopteran pests (Liu et al. 2025). Many of these diurnally active predators contribute substantially to natural pest regulation in agroecosystems (Miller 1971). Owing to their demonstrated predatory efficiency, harpactorine predators are increasingly employed in biological control programs within agricultural and forestry systems (Feng et al. 2021; Ren et al. 2022).

To date, the only formally published *Rhynocoris* mitogenome is that of *R. fuscipes*, whose complete mitogenome suggested *Rhynocoris* as sister to *Sphedanolestes* (Wang et al. 2022), while GenBank records exist for *R. incertis* and *R. marginatus* (unpublished). The lack of mitogenomic data for widespread species such as *R. altaicus* limits our understanding of the evolutionary history of this genus.

In this study, we for the first time sequenced and characterized the complete mitochondrial genome of *R. altaicus*, providing essential genomic resources for future phylogenetic and evolutionary investigations.

## Materials and methods

Specimens of *Rhynocoris altaicus* were collected from Jilin City, China (43°46′52.11408″N, 126°40′56.04042″E) in May 2023 using light traps. The voucher specimen (No. LC-202401) was photographed and deposited at the Key Laboratory of Integrated Pest Management in Ecological Forests, Fujian Province University, Fujian Agriculture and Forestry University (URL: https://lxy.fafu.edu.cn, contact person: Songqing Wu; email: dabinyang@126.com) (Figure 1).

**Figure 1.**
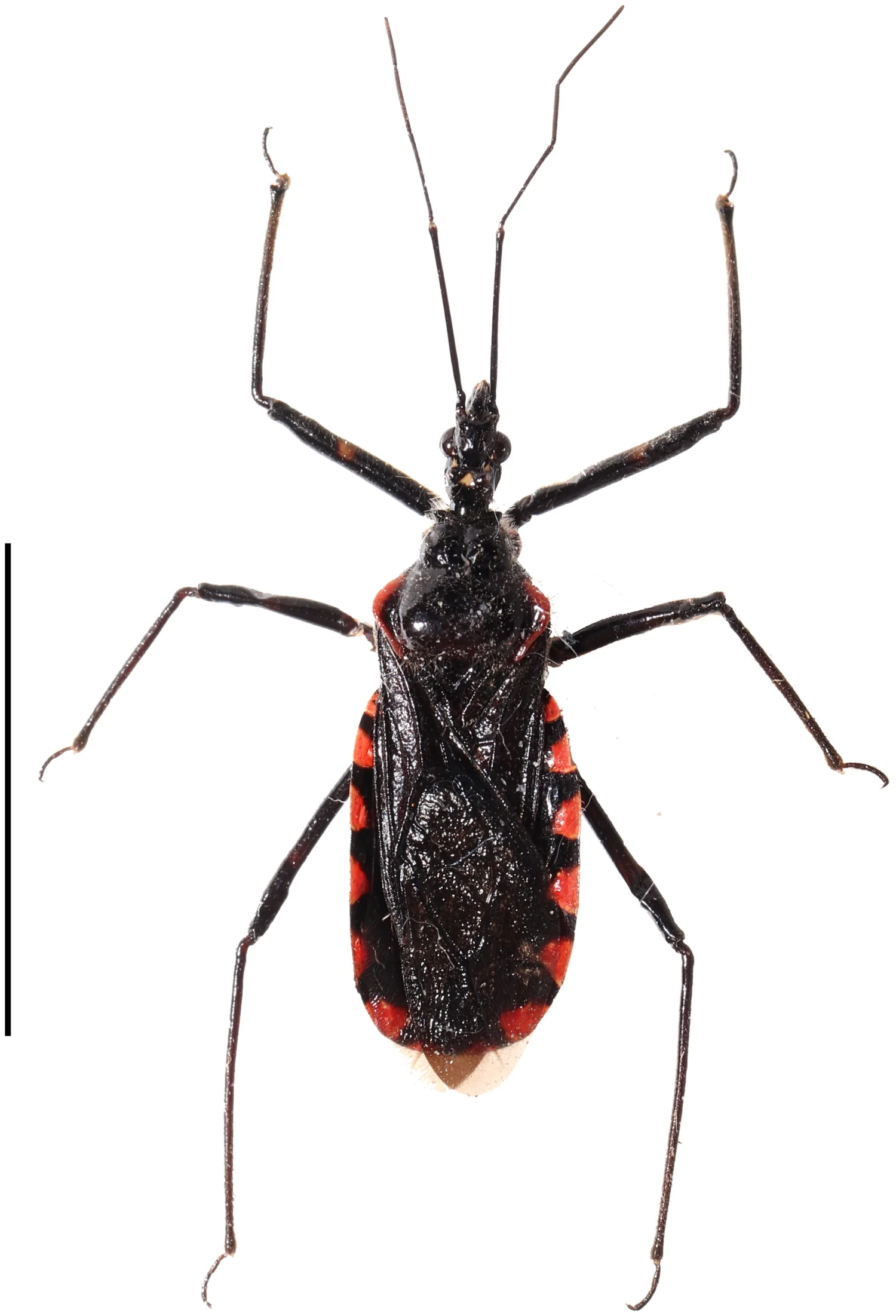
Dorsal view of specimen of *Rhynocoris altaicus* Kiritshenko, 1926, showing the single red annular marking on each femur . Photographed by Zhi-Ming Zhang. Scale bar = 1 cm.

Genomic DNA was extracted from leg muscle of an adult specimen using the TIANamp Genomic DNA Kit (Tiangen Biotech, Beijing, China) and quantified with a NanoDrop 2000 (Thermo Fisher Scientific, Waltham, MA). A sequencing library was constructed using the TruSeq Nano DNA HT Sample Prep Kit (Illumina, San Diego, CA) and sequenced on the Illumina NovaSeq 6000 platform (Illumina, San Diego, CA) with 150 bp paired-end reads. Raw reads were filtered to remove low-quality sequences, adapters, and short reads (<60 bp), yielding 1,603,330 clean reads. The mitogenome was assembled using GetOrganelle v1.7.7.1 (Jin et al. 2020) with default parameters and MitoZ v3.6 (Meng et al. 2019), and annotated with the MITOS2 webserver (Bernt et al. 2013). Tandem repeats in the D-loop and the intergenic spacer were detected using Tandem Repeats Finder (TRF; Benson 1999).

For phylogenetic analysis, the 13 protein-coding genes (PCGs) of *Rhynocoris altaicus* and other Reduviidae species were retrieved from NCBI. Multiple sequence alignment was conducted using PhyloSuite v1.2.3 (Zhang et al. 2020) (which calls MAFFT). The concatenated dataset of 13 PCGs (11,155 bp) was partitioned into seven subsets based on gene combination. The best-fit nucleotide substitution models for each partition were selected using ModelFinder (Kalyaanamoorthy et al. 2017) implemented in IQ-TREE (v2.2.0) (Minh et al. 2020) based on the Bayesian information criterion (BIC). Maximum-likelihood phylogeny was then inferred using IQ-TREE with the selected partition-specific models. Node support values were evaluated via 1000 ultrafast bootstrap replicates (Hoang et al. 2018) and 1000 SH‑aLRT tests (Guindon et al. 2010). The resulting tree was annotated using FigTree v1.4.4 (Edinburgh, UK).

## Results

The newly assembled mitochondrial genome (Figure 2) is 16,177 bp in length, with an overall A + T content of 70.2% (A = 41.0%, T = 29.2%, C = 18.1%, G = 11.7%). The mitogenome was assembled with an average sequencing coverage of ×2707 (Figure S1).

**Figure 2.**
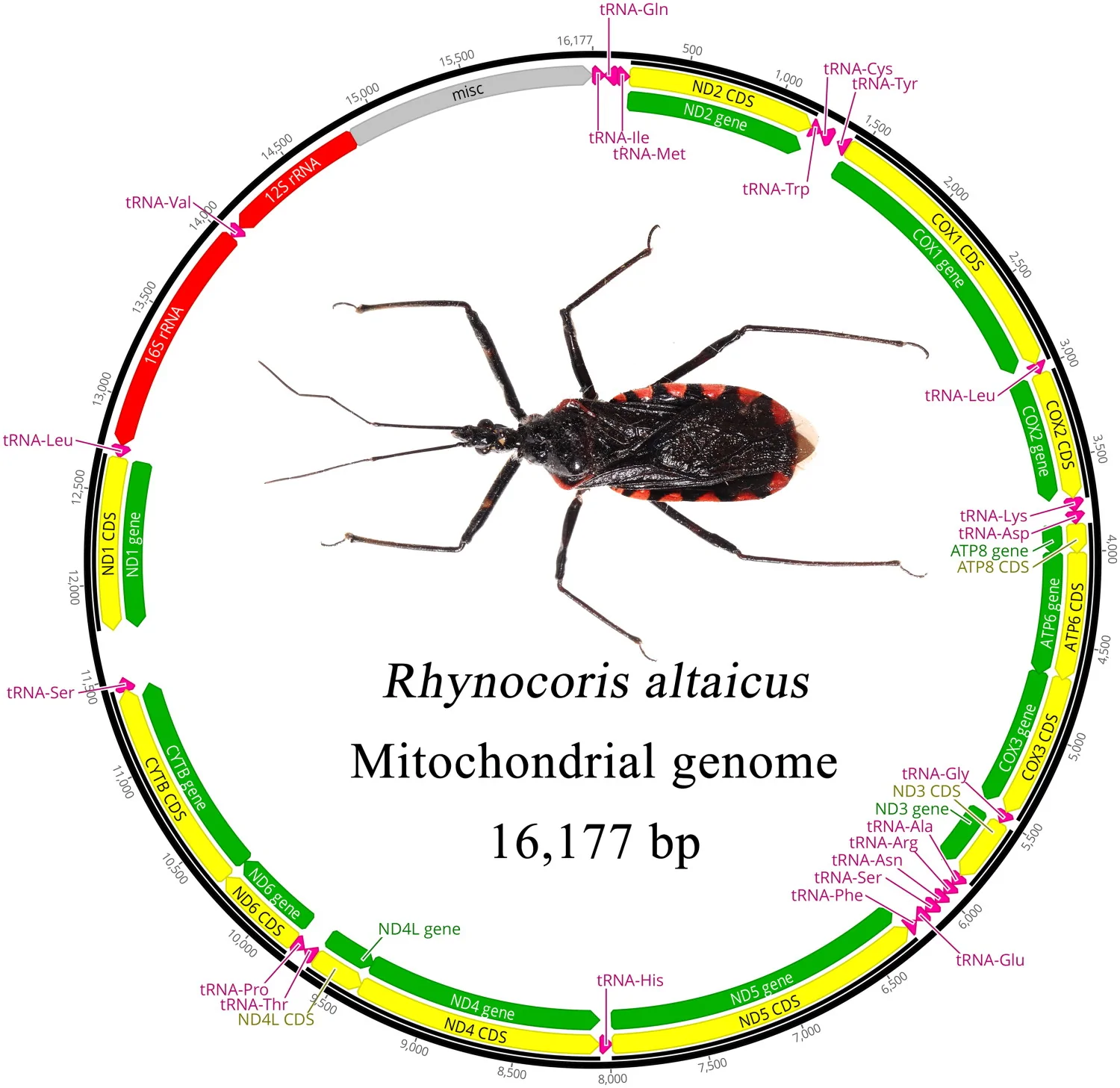
Circular map of the mitochondrial genome of *Rhynocoris altaicus* Kiritshenko, 1926. Protein-coding genes are shown as two concentric circles: the outer yellow circle indicates coding sequence and the inner green circle indicates gene regions. rRNAs are shown in red, tRNAs in magenta, and the D-loop in grey. The transcription direction is indicated by arrows.

The genome comprises 13 PCGs, 22 transfer RNA genes (tRNAs), and two ribosomal RNA genes (rRNAs) (12S rRNA and 16S rRNA), as well as a D-loop of 1326 bp. TRF analysis revealed D-loop a 214 bp tandem repeat with 2.6 copies and 99% sequence identity. A 261 bp intergenic spacer was identified in the CYTB–trnS–ND1 region (Table S1); TRF analysis detected no tandem repeats. The gene arrangement and orientation are consistent with those of other sequenced Reduviidae mitogenomes (Figure 2).

Among the 13 PCGs, four genes (ND2, ND5, ATP8, COX1) started with ATT, two genes (COX2, ND3) started with ATA, and the remaining seven PCGs (ND1, ND4, ND6, ND4L, COX3, CYTB, ATP6) started with ATG. For stop codons, six PCGs (ND1, ND2, ND6, COX1, ATP8, ATP6) terminated with TAA, four PCGs (ND3, ND5, ND4L, CYTB) terminated with TAG, and three PCGs (COX2, COX3, ND4) used an incomplete stop codon T (Table S1).

For phylogenetic reconstruction, the concatenated dataset of 13 mitochondrial PCGs was partitioned into seven subsets based on gene combination. The best-fit models under the BIC were: GTR + F + I + G4 for ATP6 + ND2, HKY + F + I + G4 for ATP8, TIM2 + F + I + G4 for COX1, GTR + F + I + G4 for COX2 + COX3, TIM2 + F + I + G4 for CYTB + ND3, TIM + F + I + G4 for ND1 + ND4L + ND4 + ND5, and TN+F + I + G4 for ND6. Maximum-likelihood phylogenetic analysis based on the concatenated dataset recovered a robust topology in which *R. altaicus* forms a well-supported clade with *R. fuscipes*, *R. incertis*, and *R. marginatus*, and the genus *Rhynocoris* was recovered as monophyletic within Harpactorinae (Figure 3).

**Figure 3.**
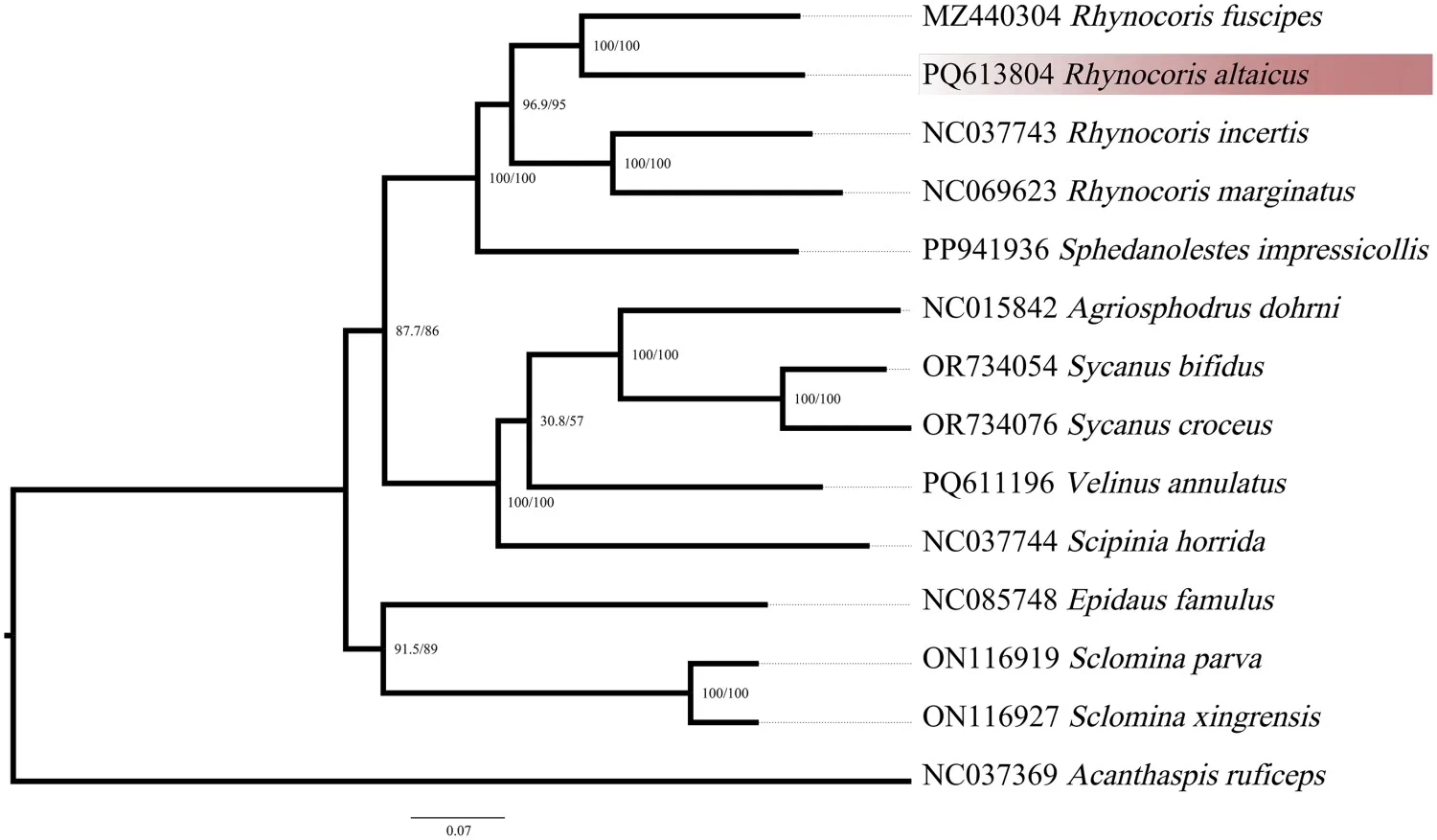
Maximum-likelihood tree based on 13 PCGs of Reduviidae species. Nodal support values are shown as SH-aLRT support/ultrafast bootstrap. The newly sequenced species is indicated in highlight. The following sequences were used: ON116919 and ON116927 (Du et al. 2023), NC015842 and OR734076 (Li et al. 2011), OR734054 (Chen et al. 2024), MZ440304 (Wang et al. 2022), PQ613804 (this study), NC085748, NC037744, PP941936, NC037743, NC069623, NC037369, and PQ611196 (unpublished).

The complete mitochondrial genome sequence has been deposited in GenBank under accession number PQ613804.

## Discussion and conclusions

This study presents the first complete mitochondrial genome of *Rhynocoris altaicus*, a common Palearctic predator with biological control potential. The mitogenome is 16,177 bp in length with typical gene content and organization, similar to other reduviid species such as *R. fuscipes* (Wang et al. 2022). Analysis of the D-loop revealed a 214 bp tandem repeat with 2.6 copies, suggesting a partially duplicated structure within this non-coding region. Additionally, a 261 bp intergenic spacer was identified in the CYTB–trnS–ND1 region. Similar long spacers have been reported in other reduviids, such as a 314 bp spacer containing tandem repeats in *Triatoma dimidiata* (Dotson and Beard 2001).

Phylogenetic analysis based on 13 PCGs placed *R. altaicus* within a well-supported clade containing *R. fuscipes*, *R. incertis*, and *R. marginatus*, confirming the monophyly of *Rhynocoris*. This result is consistent with previous findings (Wang et al. 2022) and provides improved phylogenetic resolution compared to single-gene analyses. Our phylogeny recovered *Rhynocoris* as sister to *Sphedanolestes*, consistent with the placement of Harpactorinae as monophyletic in previous studies (Masonick et al. 2025). The mitochondrial genome data obtained will facilitate future research on population genetics, molecular identification, and biological control of this ecologically important predator, which feeds mainly on lepidopteran larvae.

## Supplementary Material

supplementary material.docx

## Data Availability

The genome sequence data supporting this study’s findings are available in GenBank of NCBI at https://www.ncbi.nlm.nih.gov under assessment no. PQ613804. The associated BioProject, Bio-Sample, and SRA numbers are PRJNA1203931, SAMN46004801, and SRS23653666, respectively.
