# Supplementary material for: The complete mitochondrial genome of Rhynocoris altaicus Kiritshenko, 1926 (Hemiptera: Heteroptera: Reduviidae)
Source: Mitochondrial DNA B Resour. 2026 Sep 29;11(10):1256–9. doi: 10.1080/23802359.2026.2739028 (PMC13629767; doi:10.1080/23802359.2026.2739028)
Supplement: Supplementary file 1 [file TMDN_A_2739028_SM7861.docx]

**Table S1.** Organization of the mitochondrial genome of *Rhynocoris altaicus*.

| Feature | Size | Start Codon | Stop Codon | Intergenic Nucleotide | Strand |
| --- | --- | --- | --- | --- | --- |
| tRNA-Ile | 65 |  |  | -3 | J |
| tRNA-Gln | 69 |  |  | -1 | N |
| tRNA-Met | 70 |  |  | 0 | J |
| ND2 | 1005 | ATT | TAA | 2 | J |
| tRNA-Trp | 65 |  |  | -8 | J |
| tRNA-Cys | 68 |  |  | 33 | N |
| tRNA-Tyr | 64 |  |  | 6 | N |
| COX1 | 1542 | ATT | TAA | 5 | J |
| tRNA-Leu | 65 |  |  | 2 | J |
| COX2 | 685 | ATA | T | 0 | J |
| tRNA-Lys | 71 |  |  | -1 | J |
| tRNA-Asp | 64 |  |  | 0 | J |
| ATP8 | 159 | ATT | TAA | -7 | J |
| ATP6 | 684 | ATG | TAA | -14 | J |
| COX3 | 781 | ATG | T | 0 | J |
| tRNA-Gly | 63 |  |  | 0 | J |
| ND3 | 354 | ATA | TAG | -1 | J |
| tRNA-Ala | 62 |  |  | 4 | J |
| tRNA-Arg | 63 |  |  | 4 | J |
| tRNA-Asn | 65 |  |  | -1 | J |
| tRNA-Ser | 69 |  |  | 1 | J |
| tRNA-Glu | 65 |  |  | 1 | J |
| tRNA-Phe | 63 |  |  | -1 | N |
| ND5 | 1716 | ATT | TAG | 0 | N |
| tRNA-His | 65 |  |  | 0 | N |
| ND4 | 1330 | ATG | T | -7 | N |
| ND4L | 285 | ATG | TAG | 6 | N |
| tRNA-Thr | 66 |  |  | 0 | J |
| tRNA-Pro | 66 |  |  | 3 | N |
| ND6 | 501 | ATG | TAA | -1 | J |
| CYTB | 1137 | ATG | TAG | -2 | J |
| tRNA-Ser | 69 |  |  | 261 | J |
| ND1 | 918 | ATG | TAA | 1 | N |
| tRNA-Leu | 65 |  |  | 0 | N |
| 16S | 1266 |  |  | 0 | N |
| tRNA-Val | 69 |  |  | -1 | N |
| 12S | 772 |  |  | 0 | N |

**Figure S1.** Depth of coverage for *Rhynocoris altaicus* mitochondrial genome. X and Y axis present nucleotide position of *Rhynocoris altaicus* mitochondrial genome and coverage depth, respectively.
